# Mass Spectrometry Based Profiling and Imaging of Various Ginsenosides from *Panax ginseng* Roots at Different Ages

**DOI:** 10.3390/ijms18061114

**Published:** 2017-05-24

**Authors:** Jae Won Lee, Seung-Heon Ji, Young-Seob Lee, Doo Jin Choi, Bo-Ram Choi, Geum-Soog Kim, Nam-In Baek, Dae Young Lee

**Affiliations:** 1 Department of Herbal Crop Research, National Institute of Horticultural and Herbal Science, Rural Development Administration (RDA), Eumseong 27709, Korea; jaewon3@gmail.com (J.W.L.); jiddung205@chungbuk.ac.kr (S.-H.L.); youngseoblee@korea.kr (Y.-S.L.); cdj0105@korea.kr (D.J.C.); bmcbr@korea.kr (B.-R.C.); kimgs0725@korea.kr (G.-S.K.); 2 Department of Oriental Medicinal Materials and Processing, Kyung Hee University, Yongin 17104, Korea; nibaek@khu.ac.kr

**Keywords:** ginsenosides, *Panax ginseng*, profiling, imaging, UPLC-QTOF/MS, MALDI

## Abstract

(1) Background: *Panax ginseng* root is one of the most important herbal products, and the profiling of ginsenosides is critical for the quality control of ginseng roots at different ages in the herbal markets. Furthermore, interest in assessing the contents as well as the localization of biological compounds has been growing. The objective of this study is to carry out the mass spectrometry (MS)-based profiling and imaging of ginsenosides to assess ginseng roots at different ages; (2) Methods: Optimal ultra performance liquid chromatography coupled to quadrupole time of flight/MS (UPLC-QTOF/MS) was used to profile various ginsenosides from *P. ginseng* roots. Matrix-assisted laser desorption ionization (MALDI)-time of flight (TOF)/MS-based imaging was also optimized to visualize ginsenosides in ginseng roots; (3) Results: UPLC-QTOF/MS was used to profile 30 ginsenosides with high mass accuracy, with an in-house library constructed for the fast and exact identification of ginsenosides. Using this method, the levels of 14 ginsenosides were assessed in *P. ginseng* roots cultivated for 4, 5, and 6 years. The optimal MALDI-imaging MS (IMS) was also applied to visualize the 14 ginsenosides in ginseng roots. As a result, the MSI cross sections showed the localization of 4 ginsenoside ions ([M + K]^+^) in *P. ginseng* roots at different ages; (4) Conclusions: The contents and localization of various ginsenosides differ depending on the cultivation years of *P. ginseng* roots. Furthermore, this study demonstrated the utility of MS-based profiling and imaging of ginsenosides for the quality control of ginseng roots.

## 1. Introduction

*Panax ginseng C.A. Meyer* is one of the most important herbal products, and its root has been widely used as a constituent of traditional medicine in Korea and other countries [1]. *P. ginseng* root contains diverse bioactive compounds, and ginsenosides are the major components. Many studies have previously reported that ginsenosides show various pharmacological properties such as anti-tumor, enhanced immune system, anti-diabetes, anti-fatigue, anti-oxidative, and anti-aging effects [2,3,4,5,6]. Due to its utility, interest in the therapeutic potential of ginseng root has been growing. Moreover, it is economically critical to evaluate and control the quality of ginseng roots in the food industry and herbal markets. One of the most important issues is to evaluate the growth age of ginseng roots, and several analytical methods have been developed for this purpose [7,8,9,10]. In particular, qualitative and quantitative ginsenoside contents have been studied widely in ginseng roots at different ages [11,12]. As ginsenosides may alter according to the growth of *P. ginseng*, it is critical to analyze ginsenosides in determining the age of a ginseng root.

For the assessment of ginsenoside contents, many analytical methods have been established by using high performance liquid chromatography (HPLC) coupled to a UV detector [13,14] or an evaporative light scattering detector (ELSD) [15,16]. However, these methods have been limited to the quantification of only a few ginsenosides. Recently, electrospray ionization (ESI)-mass spectrometry (MS) has emerged as a good tool for the sensitive and selective analysis of various ginsenosides [17,18]. In particular, quadrupole time of flight (QTOF)/MS is a sensitive detector that can perform the exact mass measurement of compounds [19,20]. The ultra-performance liquid chromatography (UPLC) system with its small particle size column is also useful for the fast and high resolution separation of compounds in a complex mixture [21]. Thus, UPLC-QTOF/MS can be used for the high-throughput, comprehensive profiling of ginsenosides.

Recently, interest in assessing the contents as well as the localization of biological compounds has been growing [22,23]. ESI-MS is useful for the qualitative and quantitative analysis of ginsenosides. However, this instrument is insufficient for determining the localization of ginsenosides. Instead of this, matrix-assisted laser desorption ionization (MALDI)-imaging MS (IMS) has been widely applied for the sensitive and high-resolution imaging of biological molecules [24,25,26]. Taira et al. has previously reported on the application of MALDI-IMS for the visualization of ginsenosides present in the ginseng root [27]. This study first revealed that ginsenosides are mainly localized in the periderm and part of the cortex at the center of the root. Bai et al. also improved MALDI-IMS to visualize various ginsenosides on the lateral root of *P. ginseng*, and applied the data of ginsenosides to discriminate the *P. ginseng* at ages of 2, 4, and 6 years [28]. This indicated that the localization of ginsenosides alters depending on the growth age of *P. ginseng*.

In this study, the optimal UPLC-QTOF/MS with an in-house library was used to profile various ginsenosides from *P. ginseng* roots cultivated for 4, 5, and 6 years. We then attempted to find the differentially expressed ginsenosides (DEGs) in the *P. ginseng* roots depending on the different ages. Next, the MALDI-TOF/MS-based imaging method was constructed to localize the DEGs in the *P. ginseng* roots cultivated for 4, 5, and 6 years. Finally, we evaluated the utility of dual MS-based profiling and imaging of ginsenosides as a tool for the discrimination of *P. ginseng* roots at different ages.

## 2. Results

### 2.1. Optimal UPLC-QTOF/MS Method to Profile Various Ginsenosides

**Figure 1:**
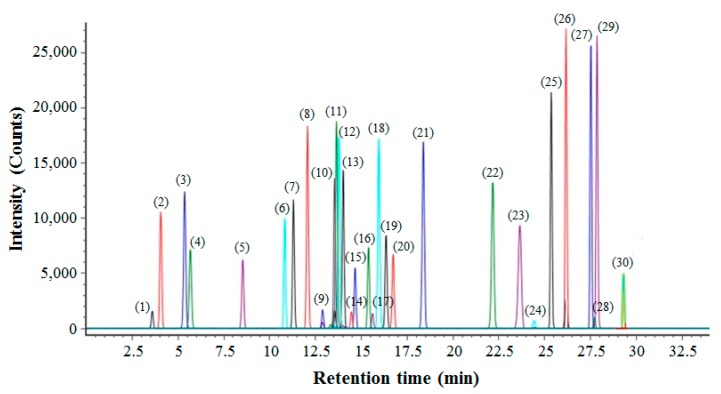
The base peak intensity (BPI) chromatogram of 30 ginsenoside standards. The peaks of (1)–(30) were equal to 30 ginsenosides, as listed in Table 1.

For the profiling of various ginsenosides from *P. ginseng* roots, the UPLC-QTOF/MS method was developed. First, 30 ginsenoside standards were used to optimize the LC/MS conditions. By using the UPLC system with an ACQUITY BEH C18 column (2.1 mm × 100 mm, 1.7 µm particle size), these standards were well separated in 30 min at a flow rate of 450 µL/min. The base peak intensity (BPI) chromatograms of 30 ginsenosides are shown in Figure 1. In the negative ion mode, each ginsenoside was detected as [M + COOH]^−^ ions with high mass accuracy (Δ < 4.5 ppm). For the fast and reliable identification of ginsenosides, we constructed an in-house library using the UNIFI software (version 1.7.1; Waters Corp., Milford, MA, USA). Information on the compound’s name, molecular formula, retention time (RT), mass accuracy, and adduct are listed in the library for 30 ginsenosides (Table 1). Second, a validation study was performed to prove the performance of the UPLC-QTOF/MS method in profiling ginsenosides. For this, the standard curves of 30 ginsenosides were constructed, and their linearity range and correlation were calculated. The limits of detection (LODs) for each standard are also listed (Table 2).

### 2.2. Profiling of Various Ginsenosides from P. ginseng Roots with Different Ages

**Figure 2:**
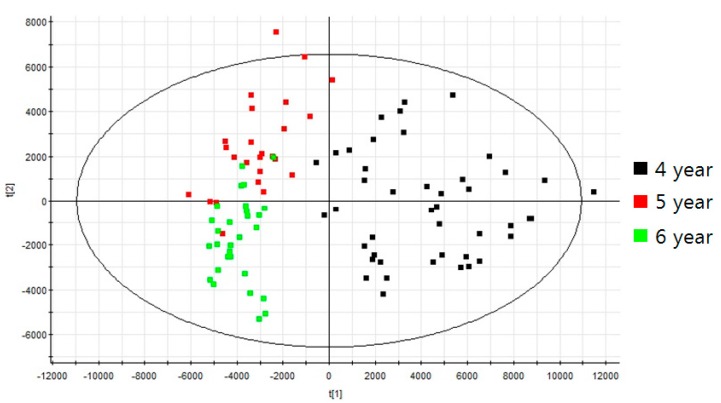
Principal component analysis (PCA) score plot of ginsenoside extracts from the ginseng roots, cultivated for 4, 5, and 6 years. 4 year roots (*n* = 44); 5 year roots (*n* = 25); 6 year roots (*n* = 29), R2X[1] = 0.2428 and R2X[2] = 0.08721.

**Figure 3:**
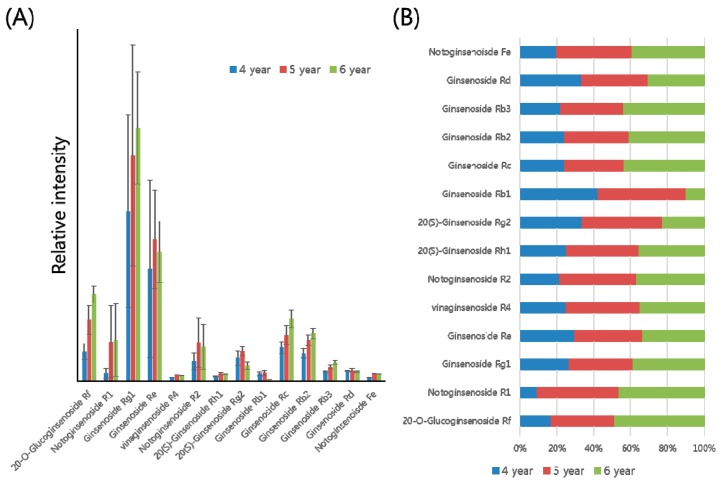
Bar plots (**A**) and percentage ratio chart (**B**) of 14 ginsenosides from *P. ginseng* roots cultivated for 4, 5, and 6 years.

The analytical platform based on UPLC-QTOF/MS with an in-house library constructed in this study was applied to profile various ginsenosides from *P. ginseng* roots at different ages. For this experiment, the main root of *P. ginseng* was used. The numbers of individual samples were as follows: 4 year roots (*n* = 44), 5 year roots (*n* = 25), and 6 year roots (*n* = 29). Then 70% (*v*/*v*) methanol was used to extract various ginsenosides from the *P. ginseng* roots. In the estimation of the extraction efficiency, the percentage of extracted ginsenoside (peak intensity of Rg3 in a ginseng extract/peak intensity of a single Rg3 × 100 (%)) was examined as about 9% (*n* = 3). The extracts were analyzed using UPLC-QTOF/MS; each piece of data was then processed using the UNIFI software. Principal component analysis (PCA) of the processed datasets was performed to visualize the clustering trends among the ginseng roots at 4, 5, and 6 years of age. In the score plot of PCA, three groups of 4, 5, and 6 year roots were well differentiated (Figure 2). The PCA loading plot also represented several ginsenosides such as Rg1, Re, and malonyl-ginsenosides as main variables to separate the three groups (Figure S1). Through data processing, a total of 14 ginsenosides were identified and quantified from the ginseng root samples. According to the different ages of ginseng roots, the bar plots showed the different levels of 14 ginsenosides (Figure 3A). To compare the quantitative levels of each ginsenoside in the three groups, we also constructed a percentage ratio chart (Figure 3B).

### 2.3. Optimal MALDI-TOF/MS-Based Imaging of Ginsenosides

**Figure 4:**
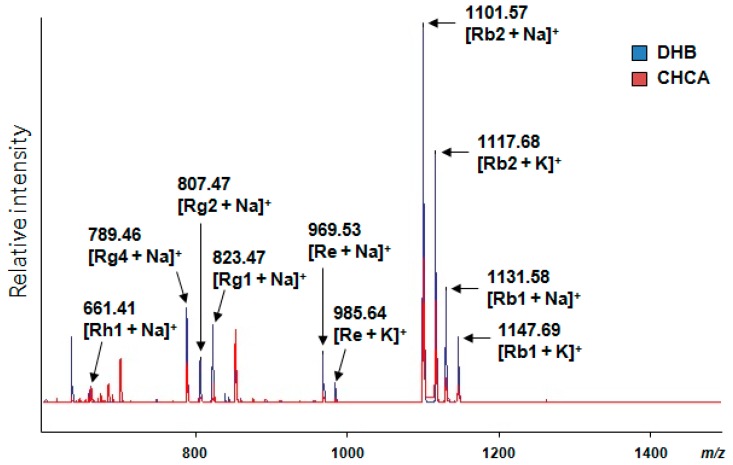
Two merged MALDI-TOF/MS spectra of seven ginsenosides (Rh1, Rg2, Rg4, Rg1, Re, Rb2, and Rb1) obtained using the 2,5-dihydroxybenzoic acid (DHB) (**blue**) and α-cyano-4-hydroxy-cinnamic acid (CHCA) (**red**) matrices, respectively. In positive ion mode, seven ginsenosides were detected with the adduction of sodium (Na) or potassium (K).

For the sensitive detection and effective localization of ginsenosides in the ginseng root, we optimized the MALDI imaging MS (IMS) conditions. To select the most effective matrix for the IMS of ginsenosides, we evaluated two commercial MALDI matrices: 2,5-dihydroxybenzoic acid (DHB) and α-cyano-4-hydroxy-cinnamic acid (CHCA). The CHCA matrix consists of 1.4 mg of α-cyano-4-hydroxy-cinnamic acid dissolved in 1 mL of solvent mixture containing 85% acetonitrile (ACN), 15% water, and 0.1% trifluoroacetic acid (TFA). In the case of the DHB matrix, DHB was prepared at a concentration of 10 mg/mL in ACN: 0.1% TFA (30:70 (*v*/*v*)) in water. In this test, a portion (1 μL) of a standard mixture including seven ginsenosides (Rh1, Rg2, Rg4, Rg1, Re, Rb2, and Rb1) and CHCA or DHB matrix mixture solution (1:1, *v*/*v*) was spotted onto the MALDI target plate (MTP 384 ground steel) (Bruker Daltonics, Bremen, Germany), and the drying of the spotted sample aliquot at room temperature was subsequently performed. Afterward, the sample was subjected to MALDI-TOF/MS analysis controlled by FlexControl version 3.4 (Bruker Daltonics, Bremen, Germany). The ginsenoside standards were detected as [M + Na]^+^ and [M + K]^+^ in the positive ion mode. The spectra were gathered manually from each matrix spot on the MALDI plate and summed and processed in baseline subtraction using FlexAnalysis version 3.4 (Bruker Daltonics, Bremen, Germany). After data processing, we compared their peak intensities obtained using the CHCA and DHB matrices. As a result, DHB provided higher ginsenoside intensity than CHCA in the MS spectrum (Figure 4). Thus, in this study, the DHB matrix was selected for the IMS of ginsenosides. Next, we optimized several conditions for sectioning the ginseng root tissue, matrix application, and MALDI-IMS analysis, as described in Materials and Methods.

### 2.4. Localization of Ginsenosides in the P. ginseng Roots at Different Ages

**Figure 5:**
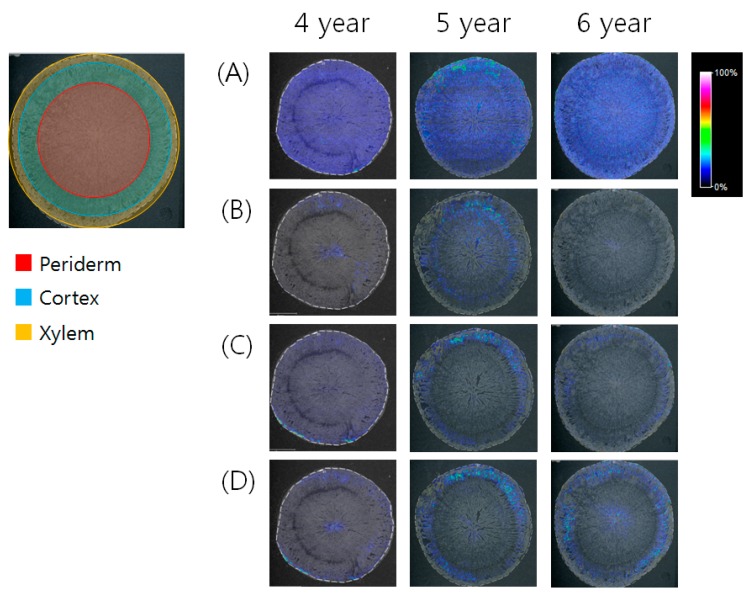
MALDI-MSI cross sections showing the localization of (**A**) Rh1 ([M + K]^+^, *m*/*z* 677.9), (**B**) Rg2 ([M + K]^+^, *m*/*z* 823.3), (**C**) Rc or Rb2 or Rb3 ([M + K]^+^, *m*/*z* 1117.5), and (**D**) Rb1 ([M + K]^+^, *m*/*z* 1147.6) from ginseng roots cultivated for 4, 5, and 6 years.

The optimal MALDI-IMS approach was applied to localize the ginsenosides in *P. ginseng* roots cultivated for 4, 5, and 6 years. From the whole body of each ginseng root, the part of the main root was applied to MS imaging. The main root of the frozen ginseng tissue was cross-sectioned, and then thaw-mounted on indium-tin-oxide (ITO)-coated glass slides (Bruker Daltonics, Bremen, Germany). The tissue sections were dehydrated by desiccators and stored at −80 °C until use. After spraying the saturated matrix solution of DHB on the tissue, the samples were subjected to MS imaging. After the acquisition of MALDI-IMS data obtained from ginseng roots cultivated for 4, 5, and 6 years, the localizations of four ginsenoside ions having *m*/*z* 677.9, 823.3, 1117.5, and 1147.6 were observed in the reconstituted MS image (Figure 5). Based on the theoretical *m*/*z* values of 14 ginsenosides differentially expressed depending on the different ages, the four ions were assigned as the [M + K]^+^ ions of Rh1 (*m*/*z* 677.9), Rg2 (*m*/*z* 823.3), Rc or Rb2 or Rb3 (*m*/*z* 1117.5), and Rb1 (*m*/*z* 1147.6). Although other ions were detected in the tissue by MALDI-IMS (Supplemental Figure S2), their *m*/*z* values were not matched with the theoretical *m*/*z* value of [M + K]^+^ or [M + Na]^+^ ions of the known ginsenosides. The MALDI-MSI cross-sections showed that each ginsenoside has a different localization according to the ages of the ginseng root.

## 3. Discussion

This study had two objectives, one of which was to develop MS-based analysis methods for the profiling and imaging of various ginsenosides from the *P. ginseng* roots. In the herbal markets, it is critical to control the quality of *P. ginseng* roots as the resource of ginsenosides. In recent decades, many pharmacological efficacies of various ginsenosides have been widely studied. The efficacies differ according to the kinds of ginsenosides. Thus, the effective use of ginsenosides requires assessing the contents and localization of ginsenosides from ginseng roots. For this purpose, the MS-based profiling and imaging method is suitable for use. The other objective is to evaluate the utility of MS-based profiling and imaging of ginsenosides for discriminating the different ages (4, 5, and 6 years old) of *P. ginseng* roots. The biosynthesis of various ginsenosides may differ depending on the growth period of ginseng roots. In particular, not only the contents but also the localization of ginsenosides may be altered with the different ages of ginseng roots. Thus, we expected that *P. ginseng* roots at different ages could be discriminated using the profiling and imaging of ginsenosides.

First, UPLC-QTOF/MS was effective in profiling various ginsenosides in a mixture. Compared to the transitional HPLC, UPLC with a small particle size column provided high throughput and effective separation of ginsenosides. As a result, 30 ginsenosides were successfully analyzed in 30 min. In particular, among them, nine isomeric pairs—C_48_H_82_O_19_ (Rf or R4), C_48_H_82_O_18_ (Re or Rd), C_42_H_72_O_14_ (Rg1 or F11), C_41_H_70_O_13_ (R2 or F5 or F3), C_58_H_98_O_26_ (Ra2 or Ra1), C_53_H_90_O_22_ (Rc or Rb2 or Rb3), C_42_H_70_O_12_ (Rg4 or F4 or Rg5), C_42_H_72_O_13_ (F2 or Rg3), and C_36_H_62_O_8_ (CK or Rh2)—were well separated from each other. QTOF/MS is also a sensitive and robust tool for measuring the exact mass values of compounds. In the mixture of the ginseng extract, there may be a variety of known/unknown compounds aside from ginsenosides. Thus, for the reliable identification of ginsenosides from the complex mixture of ginseng extract, exact mass measurement based on QTOF/MS should be used. Moreover, the in-house library constructed in this study will be useful for the fast and reliable identification of 30 ginsenosides based on RT and mass accuracy.

Optimal UPLC-QTOF/MS with an in-house library was used to profile ginsenosides from *P. ginseng* cultivated for 4, 5, and 6 years. In the whole body of the ginseng root, the ginsenoside contents may be altered depending on the different parts, such as rhizome, main, lateral, and fine roots [11]. In this study, we used only the main root of *P. ginseng* to extract ginsenosides. For estimating the extraction efficiency of ginsenosides, we used a standard (Rg3) which is absent in the used ginseng root. After the LC/MS analysis of two samples, a single Rg3 dissolved in 70% (*v*/*v*) methanol and a ginseng extract spiked with the same amount of Rg3, their peak intensities were used to calculate the extraction efficiency. This experiment assessed not only the extraction performance but also the matrix effect and the actual amounts of ginsenosides. PCA that provides an overview of groupings, trends, and outliers was applied to visualize the general clustering trends among the 4, 5, and 6 year roots. Each point of the PCA score plot shows an individual sample, and the samples are scattered by the similarities or differences of their metabolic compositions. In the score plot, three groups of 4, 5, and 6 year ginseng roots were well discriminated (Figure 2). This indicated that the components of *P. ginseng* root extracts alter depending on the growth ages. In particular, several ginsenosides were identified as the main variables in the PCA loading plot (Figure S1). Hence, it was found that ginsenosides profiling has the potential to discriminate the growth ages of the *P. ginseng* root. In the data processing, a total of 14 ginsenosides were analyzed in the main root of *P. ginseng* cultivated for 4, 5, and 6 years. From the 30 ginsenosides listed in the in-house library, the 14 species analyzed here were Rf, R1, Rg1, Re, R4, R2, Rh1, Rg2, Rb1, Rc, Rb2, Rb3, Rd, and Fe as shown in Figure 3. Among them, the levels of Rf, R1, Rg1, Rc, Rb2, and Rb3 were highest in roots cultivated for 6 years. In the case of Re, R4, R2, Rh1, Rg2, Rb1, Rd, and Fe, their levels were highest in roots cultivated for 5 years. Most ginsenosides increased in roots cultivated for 5 and 6 years, except for Rb1 and Rg2. These results indicated that the quantitative contents of ginsenisides were correlated with the growth ages of *P. ginseng*. Thus, the ratio of multiple ginsenosides can be applied to discriminate the ages of *P. ginseng* roots.

Next, we applied the optimal MALDI-IMS for the detection and localization of differentially expressed ginsenosides in *P. ginseng* roots cultivated for 4, 5, and 6 years. In the MALDI-IMS analysis, it is important to use a suitable matrix. For the MS imaging of ginsenosides, CHCA was used as a matrix in a previous study [27]. To select the most effective matrix for the MS imaging of ginsenosides, we compared the efficiency of both CHCA and DHB in detecting several standards. Although all of the compounds were well-detected by using both matrices, DHB provided a higher peak intensity than CHCA. Thus, we applied DHB for the MALDI-IMS of ginsenosides. The localization of synthesized ginsenosides may alter according to the growth period of ginseng roots. In the cross-sectioned main root, there are three distinct regions: xylem, cortex, and periderm. Among these regions, the MALDI-MSI cross sections showed the localizations of ginsenosides from *P. ginseng* roots cultivated for 4, 5, and 6 years. Compared to 4- and 6-year-old roots, Rh1 ([M + K]^+^, *m*/*z* 677.9) was only detected in the periderm of a 5-year-old root (Figure 5A). An ion with *m*/*z* 823.3 can be assigned as Rg2 based on the ginsenosides’ profiling data. Rg2 ([M + K]^+^, *m*/*z* 823.3) was detected in the xylem of a 4-year-old root as well as the cortex of a 5-year-old root (Figure 5B). An ion with *m*/*z* 1117.5 can be Rc or Rb2 or Rb3 because these ginsenosides were profiled by UPLC-QTOF/MS. Although UPLC-QTOF/MS distinguished Rc, Rb2, and Rb3 with different RTs, MALDI-IMS had limitations and assigned them only with *m*/*z* values. This ion was detected in the periderm of a 4-year-old root and the cortex of 5- and 6-year-old roots (Figure 5C). Rb1 ([M + K]^+^, *m*/*z* 1147.6) was also detected in the xylem of a 4-year-old root as well as the cortex of 5- and 6-year-old roots (Figure 5D). These results showed that several ginsenosides were differentially localized depending on the different ages of ginseng roots. It is interesting that the growth ages (4, 5, and 6 years) affected the distribution of ginsenosides in the xylem, cortex, and periderm. This may assist in studying the metabolism of ginsenosides and related enzymes as well as the discrimination of *P. ginseng* roots at different ages. MALDI-IMS is a robust tool to find the spatial distribution of various components and their relative abundance. It is also applicable for understanding the metabolism of herbal plants. The localization of ginsenosides can contribute to not only the detail phenotype of *P. ginseng* root but also to the effective usage of ginsenosides. In the future, this MS-based imaging method will be useful for characterizing many other herbal plants and for studying their active ingredients.

In conclusion, the quantitative contents and localizations of ginsenosides in *P. ginseng* roots were altered with their different growth ages. Furthermore, this study showed the utility of dual MS-based profiling and imaging of ginsenosides to discriminate *P. ginseng* roots at different ages. This method can be a good tool for not only the quality control of *P. ginseng* roots but also for the study of ginsenoside biosynthesis.

## 4. Materials and Methods

### 4.1. Standard Constituents and Reagents

HPLC-grade methanol (MeOH), water, and ACN were purchased from Fisher Scientific Korea (Seoul, Korea). HPLC-grade formic acid was purchased from Fluka Chemie GmbH (Buchs, Switzerland). TFA was purchased from Sigma-Aldrich (St. Louis, MO, USA). DHB, CHCA Matrix for MALDI-TOF MS, Peptide Calibration Standard II, microtiter plate (MTP) 384 target plate ground steel BC and MTP Slide Adapter II were purchased from Bruker Daltonics (Bremen, Germany).

Standard compounds were isolated and purified from *P. ginseng* roots and red ginseng by a series of chromatography procedures in our laboratory, and their structures were elucidated by comparing the spectroscopic data (MS, ^1^H-NMR, and ^13^C-NMR) with the literature data: 20-*O*-Glucoginsenoside Rf [29], notoginsenoside R1 [30], R2 [30], Fe [31], vinaginsenoside R4 [32], pseudo-ginsenoside F11 [33], ginsenoside Rh6 [34], F5 [35], F3 [35], Ra1 [36], Ra2 [36], F1 [37], Rh1 [38], Rg4 [39], F4 [39], F2 [38], Rg5 [40], and Rh2 [41]. Ginsenoside Rg1, Re, Rg2, Rb1, Rc, Rb2, Rb3, and Rd standards were purchased from Chromadex (Irvine, CA, USA), and ginsenoside 20(S)-Rg2 and CK were obtained from the Ambo Institute (Seoul, Korea). Rh3 and Rh21 were kindly provided by NPCL, Kyung Hee University (Suwon, Korea). The purity of the isolated compounds was determined (>98%) by the normalization of peak areas in the HPLC analysis.

### 4.2. Panax Ginseng Sample

*P. ginseng* roots were cultivated in the experimental field of Kyung Hee University located in Gangwon Province, according to the protocol of the “ginseng GAP standard cultivation guide” developed by the Korea Tobacco & Ginseng Corporation, Republic of Korea. Four-, five-, and six-year-old ginseng roots were harvested in 2014. Voucher specimen (NIHHS141010) was deposited at the herbarium of the Department of Herbal Crop Research, NIHHS, RDA, Eumseong, Republic of Korea.

### 4.3. Extraction of Ginsenosides

Each sample was dried at 40 °C in a forced-air, convection-drying oven for 48 hours after washing, and was then weighed. The main roots were used for experiments after removing the lateral and fine roots. The roots were ground (<0.5 mm) using a mixer (Hanil, Seoul, Korea) and thoroughly mixed, and subsamples were homogenized further using a Retsch MM400 mixer mill (Retsch GmbH, Haan, Germany) for analyses. Fine powder was weighed (200 mg), suspended in 2 mL of 70% (*v*/*v*) methanol, and ultrasonically extracted for 30 min at 50 °C. For solid phase extraction (SPE), Sep-Pak C18 cartridges were eluted slowly using 3 mL MeOH for the lower conditioning, and then using 3 mL dd-H_2_O for the upper conditioning. One mL of the extracted solution (50% MeOH extract) was loaded into the cartridge and eluted slowly using 10 mL dd-H_2_O to remove any sugar soluble materials, then eluted slowly using 2 mL MeOH to extract the crude ginsenosides. The final eluent was filtered and evaporated in a vacuum, and the residue was dissolved in 70% methanol. The solution was filtered through a syringe filter (0.22 µm) and injected directly into the UPLC system.

### 4.4. UPLC-QTOF/MS Conditions for Ginsenosides Profiling

UPLC was performed using a Waters ACQUITY H-Class UPLC (Waters Corp., Milford, MA, USA) with an ACQUITY BEH C18 column (2.1 mm × 100 mm, 1.7 µm). The column oven was set as 40 °C, and the mobile phases consisted of solvent A (Water + 0.1% formic acid (*v*/*v*)) and solvent B (Acetonitrile + 0.1% formic acid (*v*/*v*)). The elution gradient was as follows: 0–0.5 min, B 15%; 0.5–1 min, B 15–20%; 1–6 min, B 20%; 6–13 min, B 20–30%; 13–23 min, B 30–35%; 23–24 min, B 35–38%; 24–27 min, B 38–60%; 27–31 min, B 60–90%; 31–32 min, B 90–15%; 32–35 min, B 15%. The flow rate was 450 μL/min, and the injection volume was 2 μL for each run.

Next, MS analysis was performed using a Waters Xevo G2-S QTOF MS (Waters Corp., Milford, MA, USA) operating in negative ion mode. The mass spectrometers performed the alternative high- and low-collision energy scans (MS^E^ mode) to obtain data independent acquisition. The operating parameters were set as follows: cone voltage, 40 V; capillary, 3.0 kV; source temperature, 120 °C; desolvation temperature, 550 °C; cone gas flow, 30 L/h; and desolvation gas flow, 800 L/h. Accurate mass measurements were performed by using an automated calibration delivery system containing the internal reference [Leucine-enkephalin, *m*/*z* 554.262 (ESI−)]. Data were collected between 100 and 2000 *m*/*z*.

### 4.5. Data Processing and Multivariate Analysis

All MS^E^ data were collected and processed using UNIFI 1.8 (Waters Corp., Milford, MA, USA). Data within UNIFI 1.8 is passed through the apex peak detection and alignment processing algorithms. The intensity of each ion was normalized with respect to the total ion count to generate a data matrix having RT, *m*/*z* value, and the normalized peak area. Charged species, salt adducts, and fragments are all automatically aligned and grouped. The three-dimensional data including peak number (RT-*m*/*z* pair), sample name, and normalized peak areas were exported to the EZinfo software 3.0.3 (UMETRICS, Umea, Sweden) for PCA. The data were mean-centered and Pareto-scaled prior to PCA.

### 4.6. Ginseng Root Tissue Sectioning

The frozen ginseng root tissue was sectioned to a thickness of 50 μm using a cryo-cut microtome (Leica, Nussloch, Germany) at −20 °C. The optimum cutting temperature polymer (OCT) can degrade signals from IMS; special care should be taken to avoid OCT fixing on sections when the ginseng root tissue was stabilized by it. The frozen tissue sections were then thaw-mounted on ITO-coated glass slides (Bruker Daltonics, Bremen, Germany), dried in desiccators, and stored at −80 °C until use.

### 4.7. Sample Preparation and Matrix Application for MS Imaging

To obtain the optimum results, the application of MALDI matrix was performed as soon as possible after ginseng tissue sectioning. A thin section (typically 50 μm thick) of ginseng tissue was cut from fresh ginseng and mounted directly onto the ITO-coated glass slide (Bruker Daltonics, Bremen, Germany)-compatible MTP Slide Adapter II (Bruker Daltonics, Bremen, Germany) for IMS. The mounted ginseng tissue sections were placed in a vacuum desiccator for at least 30 min, and then were deposited with the matrix dissolved in acidified organic solution. This can be achieved manually through the use of a vibrational sprayer system. A total of 3 mL of saturated matrix solution of DHB (30 mg/mL) dissolved in 50% methanol/50% water/0.2% TFA was applied on the surface of the ginseng tissue sections by a commercially robotic liquid dispensing device called ImagePrep (Bruker Daltonics, Bremen, Germany). The homogeneous crystallization of the matrix on ginseng tissue was done manually using the following parameters: number of sprays during 1 cycle set to 50, 25–30% power; modulation of 10%; spray on 1 s; incubation time of 10 s; and time of drying set to 30 s. Ginseng tissue-sectioned, ITO-coated glass slides were then stored for 1 h in a vacuum desiccator in the dark until analysis. Finally, the MTP slide adapter II mounted with the ginseng tissue-sectioned ITO slide was directly transferred to MALDI-TOF/MS.

### 4.8. MALDI-IMS Conditions for Ginsenoside Imaging

MALDI-IMS was performed using an UltrafleXtreme MALDI-TOF/MS (Bruker Daltonics, Bremen, Germany) operated in the positive ion mode, reflector mode at lateral resolution of 100 μm, using 500 laser shots per spot with a frequency of 200 Hz and 100 μm pixel size. First of all, each ginseng tissue section was digitally scanned using a digital slide scanner equipped with a dual lens system (Epson Perfection V700 Photo Scanner, Epson, Shiojiri, Japan). The scanned histology ginseng tissue images were co-registered to the MSI data sets in FlexImaging version 4.0 (Bruker Daltonics, Bremen, Germany). FlexControl version 3.4 (Bruker Daltonics, Bremen, Germany) was used to perform data acquisition, and ginsenosides were visualized with FlexImaging version 4.0 to compare the differences in MS intensity and distributions of ginsenosides between ginseng tissues. Spectra were recorded in the *m*/*z* range from 600–2000 Da, and all the experiments were independently conducted in triplicate (*n* = 3). The instrument was calibrated prior to every image acquisition using a standard mixture of peptides (Peptide Calibration Standard II) including Angiotensin II (1046.542 Da), Angiotensin I (1296.685 Da), Substance P (1347.735 Da), Bombesin (1619.822 Da), ACTH clip (2093.086 Da), ACTH clip (2465.198 Da), and Somatostatin (3147.471 Da) spotted onto a serial ginseng tissue section mounted on the same ITO slide. Peak assignment tolerance was set to 500 ppm.

## Figures and Tables

**Table 1:** In-house library for the analysis of 30 ginsenosides using UPLC-QTOF/MS.

No.	RT (min)	Ginsenosides	Molecular Formula	Expected Neutral Mass (Da)	Observed Neutral Mass (Da)	QTOF/MS (ESI−) *m/z*	Mass Accuracy (ppm)	Adducts
1	3.61	20-*O*-glucoginsenoside Rf (Rf)	C_48_H_82_O_19_	962.545	962.5428	1007.541	2.2	+HCOO
2	4.07	Notoginsenoside R1 (R1)	C_47_H_80_O_18_	932.5345	932.5336	977.5318	0.9	+HCOO
3	5.38	Ginsenoside Rg1 (Rg1)	C_42_H_72_O_14_	800.4922	800.4905	845.4887	2	+HCOO
4	5.69	Ginsenoside Re (Re)	C_48_H_82_O_18_	946.5501	946.5483	991.5465	1.9	+HCOO
5	8.54	Ginsenoside Rh6 (Rh6)	C_36_H_62_O_11_	670.4292	670.4266	715.4248	3.6	+HCOO
6	10.82	Ginsenoside Rh21 (Rh21)	C_37_H_64_O_10_	668.4499	668.4478	713.4460	3	+HCOO
7	11.29	Vinaginsenoside R4 (R4)	C_48_H_82_O_19_	962.5450	962.5433	1007.5415	1.8	+HCOO
8	12.06	Pseudo-ginsenoside F11 (F11)	C_42_H_72_O_14_	800.4922	800.4897	845.4879	2.9	+HCOO
9	12.9	Notoginsenoside R2 (R2)	C_41_H_70_O_13_	770.4816	770.4783	815.4765	4.1	+HCOO
10	13.55	Ginsenoside F5 (F5)	C_41_H_70_O_13_	770.4816	770.4809	815.4791	0.9	+HCOO
11	13.64	20(S)-ginsenoside Rh1 (Rh1)	C_36_H_62_O_9_	638.4394	638.4370	683.4352	3.4	+HCOO
12	13.77	20(S)-ginsenoside Rg2 (Rg2)	C_42_H_72_O_13_	784.4973	784.4967	829.4949	0.7	+HCOO
13	14.02	Ginsenoside F3 (F3)	C_41_H_70_O_13_	770.4816	770.4806	815.4788	1.3	+HCOO
14	14.45	Ginsenoside Ra2 (Ra2)	C_58_H_98_O_26_	1210.6346	1210.6347	1255.6329	0.1	+HCOO
15	14.63	Ginsenoside Rb1 (Rb1)	C_54_H_92_O_23_	1108.6029	1108.6036	1153.6018	0.5	+HCOO
16	15.36	Ginsenoside Rc (Rc)	C_53_H_90_O_22_	1078.5924	1078.5920	1123.5902	0.3	+HCOO
17	15.58	Ginsenoside Ra1 (Ra1)	C_58_H_98_O_26_	1210.6346	1210.6332	1255.6314	1.1	+HCOO
18	15.94	Ginsenoside F1 (F1)	C_36_H_62_O_9_	638.4394	638.4386	683.4368	1.2	+HCOO
19	16.36	Ginsenoside Rb2 (Rb2)	C_53_H_90_O_22_	1078.5924	1078.5926	1123.5908	0.2	+HCOO
20	16.71	Ginsenoside Rb3 (Rb3)	C_53_H_90_O_22_	1078.5924	1078.5915	1123.5897	0.8	+HCOO
21	18.38	Ginsenoside Rd (Rd)	C_48_H_82_O_18_	946.5501	946.5499	991.5481	0.2	+HCOO
22	22.1	Notoginsenoisde Fe (Fe)	C_47_H_80_O_17_	916.5396	916.5377	961.5359	1.9	+HCOO
23	23.63	Ginsenoside Rg4 (Rg4)	C_42_H_70_O_12_	766.4867	766.4849	811.4831	2.3	+HCOO
24	24.47	Ginsenoside F4 (F4)	C_42_H_70_O_12_	766.4867	766.4851	811.4833	2	+HCOO
25	25.37	Ginsenoside F2 (F2)	C_42_H_72_O_13_	784.4973	784.4952	829.4934	2.5	+HCOO
26	26.17	Ginsenoside Rg3 (Rg3)	C_42_H_72_O_13_	784.4973	784.4956	829.4938	2	+HCOO
27	27.53	Compound K (CK)	C_36_H_62_O_8_	622.4445	622.4422	667.4404	3.3	+HCOO
28	27.72	Ginsenoside Rg5 (Rg5)	C_42_H_70_O_12_	766.4867	766.4846	811.4828	2.6	+HCOO
29	27.85	Ginsenoside Rh2 (Rh2)	C_36_H_62_O_8_	622.4445	622.4431	667.4413	2	+HCOO
30	29.3	Ginsenoside Rh3 (Rh3)	C_36_H_60_O_7_	604.4339	604.4338	649.4320	0.1	+HCOO

**Table 2:** Validation study for the analysis of 30 ginsenosides using UPLC-QTOF/MS.

Ginsenosides	Correlation (*R*^2^)	Linear Range (pg) *	LOD (pg) *	Ginsenosides	Correlation (*R*^2^)	Linear Range (pg) *	LOD (pg) *
Rf	0.9947	70–2000	70	Rc	0.9963	200–4000	200
R1	0.9997	9–2000	9	Ra1	0.9985	70–8000	70
Rg1	0.9992	12–4000	12	F1	0.9969	5–2000	5
Re	0.9840	10–12,000	10	Rb2	0.9969	2–4000	2
Rh6	0.9999	8–400	8	Rb3	0.9999	4–2000	4
Rh21	0.9824	2–160	2	Rd	0.9964	6–4000	6
R4	0.9999	4–20	4	Fe	0.9900	10–200	10
F11	0.9845	4–2000	4	Rg4	0.9984	20–1000	20
R2	0.9991	40–4000	40	F4	0.9990	200–2000	200
F5	0.9977	4–1000	4	F2	0.9998	1–10	1
Rh1	0.9998	4–2000	4	Rg3	0.9964	2–120	2
Rg2	0.9969	5–400	5	CK	0.9999	4–180	4
F3	0.9923	6–2000	6	Rg5	0.9987	20–2000	20
Ra2	0.9995	200–7200	200	Rh2	0.9997	4–1000	4
Rb1	0.9906	3–7400	3	Rh3	0.9983	16–100	16

* On-column amounts; LOD, Limits of detection.

## Supplementary Materials

Supplementary materials can be found at www.mdpi.com/1422-0067/18/6/1114/s1.

## Abbreviations

UPLC	Ultra performance liquid chromatography
QTOF	Quadrupole time of flight
ESI	Electrospray ionization
MALDI	Matrix-assisted laser desorption ionization
MS	Mass spectrometry
IMS	Imaging mass spectrometry
LOD	Limits of detection
RT	Retention time
PCA	Principal component analysis
