# Supplementary material for: Mass Spectrometry Based Profiling and Imaging of Various Ginsenosides from Panax ginseng Roots at Different Ages
Source: Int J Mol Sci. 2017 May 24;18(6):1114. doi: 10.3390/ijms18061114 (PMC5485938; doi:10.3390/ijms18061114)
Supplement: Supplementary file 1 [file ijms-18-01114-s001.pdf]

Supplementary File:

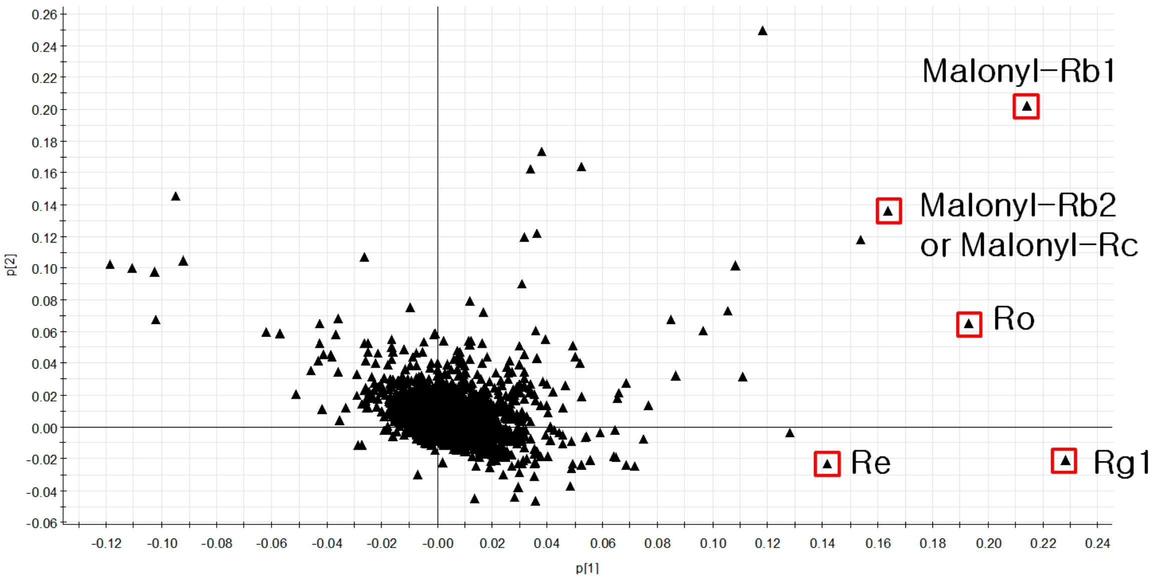

Figure S1. Principal component analysis loading plot of ginsenoside extracts from the ginseng roots, cultivated for 4, 5, and 6 years. Several points, the main variables having *m/z*_RT pairs, were identified as follows: *m/z* 845.489_5.38 (Rg1), *m/z* 955.489_15.82 (Ro), *m/z* 991.546_5.69 (Re), *m/z* 1193.594_15.35 (malonyl-Rb1), and *m/z* 1163.583_17.23 (malonyl-Rb2 or malonyl-Rc).

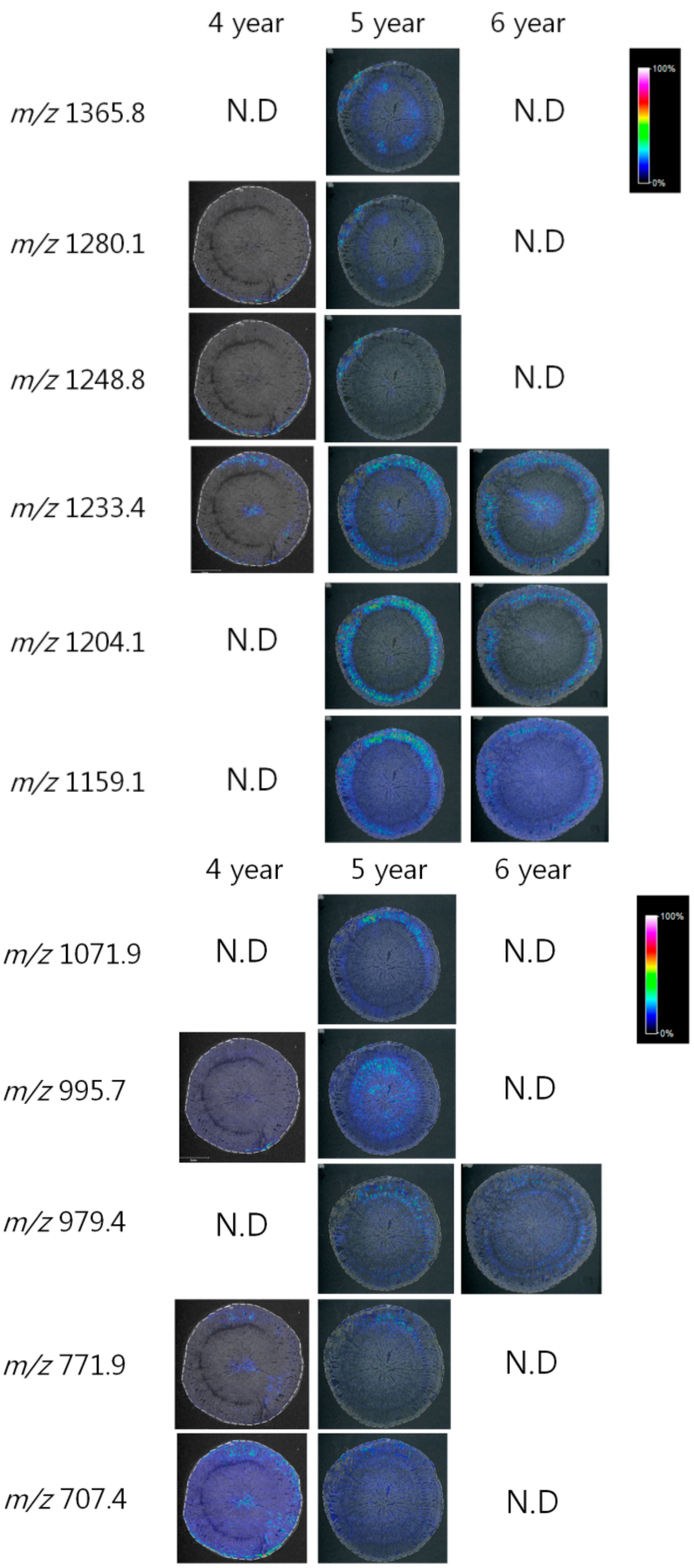

Figure S2. MALDI-MSI cross sections showing the localization of unknown ions having different *m*/*z* values (*m*/*z* 1365.8, *m*/*z* 1280.1, *m*/*z* 1248.8, *m*/*z* 1233.4, *m*/*z* 1204.1, *m*/*z* 1159.1, *m*/*z* 1071.9, *m*/*z* 995.7, *m*/*z* 979.4, *m*/*z* 771.9, *m*/*z* 707.4) from ginseng roots cultivated for 4, 5, and 6 years.
